# Identification of an HSP90 modulated multi-step process for ERBB2 degradation in breast cancer cells

**DOI:** 10.18632/oncotarget.13392

**Published:** 2016-11-16

**Authors:** Patrizio Castagnola, Grazia Bellese, Filippo Birocchi, Maria Cristina Gagliani, Carlo Tacchetti, Katia Cortese

**Affiliations:** ^1^ Dipartimento di Medicina Sperimentale, Anatomia Umana, Università di Genova, Italy; ^2^ Dipartimento di Terapie Oncologiche Integrate, IRCCS AOU San Martino – IST, Genova, Italy; ^3^ Centro Imaging Sperimentale, IRCCS Istituto Scientifico San Raffaele, Milano, Italy

**Keywords:** ERBB2, geldanamycin (GA), polyubiquitin, proteasome, cleavage

## Abstract

The receptor tyrosine kinase ERBB2 interacts with HSP90 and is overexpressed in aggressive breast cancers. Therapeutic HSP90 inhibitors, i.e. Geldanamycin (GA), target ERBB2 to degradation. We have previously shown that HSP90 is responsible for the missorting of recycling ERBB2 to degradation compartments. In this study, we used biochemical, immunofluorescence and electron microscopy techniques to demonstrate that in SKBR3 human breast cancer cells, GA strongly induces polyubiquitination and internalization of the full-length p185-ERBB2, and promotes its cleavage, with the formation of a p116-ERBB2 form in EEA1-positive endosomes (EE). p116-ERBB2 corresponds to a non-ubiquitinated, signaling-impaired, membrane-bound fragment, which is readily sorted to lysosomes and degraded. To define the sequence of events leading to p116-ERBB2 degradation, we first blocked the EE maturation/trafficking to late endosomes/lysosomes with wortmannin, and found an increase in GA-dependent formation of p116-ERBB2; we then inhibited the proteasome activity with MG-132 or lactacystin, and observed an efficient block of p185-ERBB2 cleavage, and its accumulation in EE, suggesting that p185-ERBB2 polyubiquitination is necessary for proteasome-dependent p116-ERBB2 generation occurring in EE. As polyubiquitination has also been implicated in autophagy-mediated degradation of ERBB2 under different experimental conditions, we exploited this possibility and demonstrate that GA strongly inhibits early autophagy, and reduces the levels of the autophagy markers atg5-12 and LC3-II, irrespective of GA-induced ERBB2 polyubiquitination, ruling out a GA-dependent autophagic degradation of ERBB2. In conclusion, we propose that HSP90 inhibition fosters ERBB2 polyubiquitination and proteasome-dependent generation of a non-ubiquitinated and inactive p116-ERBB2 form in EE, which is trafficked from altered EE to lysosomes.

## INTRODUCTION

ERBB2 is a member of the epidermal growth factor (EGF) receptor (ERBB1) family and is chaperoned by HSP90; unlike the other ErbB members (ERBB1, ERBB3 and ERBB4), ERBB2 has no soluble ligands. Upon activation ErbB receptors homo- or hererodimerize and ERBB2 is the preferred dimerization partner, constituting a potent signaling module [1–3]. In normal epithelial cells, dimerized ErbB receptors activate AKT and ERK-dependent pathways that govern cell proliferation and survival [3]. ERBB2 is amplified and overexpressed in 20% to 30% of human breast cancers, and it is often associated with aggressive disease and poor prognosis [4]. The oncogenic potential of ERBB2 is also linked to preferential recycling and to poor endocytic rate and traffic to lysosomes, compared to other members of the EGFR family [5]. Although the molecular mechanisms protecting ERBB2 from downregulation are not yet sufficiently explored, the hyper-stability of ERBB2 at the plasma membrane of overexpressing cells appears to be in part ensured by the interaction with the HSP90 chaperone [6–9], accompanied by lack of specific endocytic motifs in the ERBB2 sequence [10–12].

Targeted therapy based on humanized antibodies to ERBB2, i.e. trastuzumab (TZ, Herceptin®), the recently FDA-approved antibody-drug conjugate (ADCs) TZ-TDM1 (Kadcyla®, Genetech, San Francisco), or antibody-nanoparticles conjugates (ANPs), are under intense clinical investigations, in association with HSP90 inhibitors [13]. Indeed, the Geldanamycin (GA) analog 17-AAG combined with TZ and TZ-TDM1 showed clinical efficacy in ERBB2^+^ breast cancers reporting tumor progression when treated with TZ alone [14–18], suggesting that it may represent an effective strategy to overcome, or prevent drug resistance. In particular, HSP90 inhibition facilitated the intracellular delivery of TZ-conjugated nanoparticles (ANPs), carrying the chemotherapeutic drug doxorubicin, into ERBB2^+^ breast cancer cells, resulting in improved antitumor activity [18].

GA impairs the binding of ADP/ATP to HSP90 and it is widely utilized in *in vitro* studies to disrupt ERBB2 association with HSP90 [17, 19]. Indeed, HSP90 inhibitors, including GA, down-regulate ERBB2 very efficiently in several breast cancer cell lines [15, 20–28]. However, the exact mechanism whereby GA induces ERBB2 degradation is not completely understood. Early studies showed that GA potentiates ERBB2 cleavage in NIH3T3 cells that express the chimeric EGF receptor containing the ERBB2 cytoplasmic domain (EGFR/ErbB-2^CD^), originating a trans-membrane fragment of about 135kDa [29, 30]. Whether ERBB2 fragment/s are formed in ERBB2 overexpressing breast cancer cells, the nature of the protease/s involved, the cell site of the cleavage, and the possible intracellular fate of this/these fragment/s, remain unclear.

Recent studies focusing on ERBB2 internalization/trafficking showed that ERBB2 overexpression exerts a negative control on clathrin-coated pit formation [31], and on EGF-induced clathrin-coated pits [32, 33]. However, GA treatment has been reported to promote ERBB2 internalization through a clathrin- and a dynamin-dependent pathway [31, 34–36]. Moreover, GA is responsible for the missorting of the internalized ERBB2, from recycling to degradative compartments [31, 34]. Therefore, it was no surprise that GA-mediated inhibition of HSP90 was also able to trigger the recruitment of the ubiquitin ligase CHIP and/or c-cbl and to induce rapid ubiquitination of ERBB2 [7, 23, 37].

However, to make the story more complex, the polyubiquitination of ERBB2 was proposed to either induce proteasomal degradation of ERBB2 [38], to enable internalization and lysosome degradation [39], or to promote a selective autophagy of ERBB2 in breast cancer cells [6]. The exact role of the proteasome in GA-mediated ERBB2 down-regulation represents a further enigmatic issue. It has been described that the proteasome activity is required for the initial internalization step or, in contrast, for ERBB2 trafficking from early to late/lysosome compartments. These discrepancies might depend on different experimental settings and/or off-targets effects of proteasome inhibitors [35, 36, 38].

In this work, we sought to clarify whether GA induces ERBB2 cleavage in SKBR3 cells, and to characterize the ERBB2 cleaved isoform/s in terms of downstream signaling, protease/s involved and final intracellular fate. We show that GA induces polyubiquitination of the full length p185-ERBB2, and potentiates the formation of a p116-ERBB2 non-ubiquitinated and signaling impaired fragment in altered early endosomes (EE), which is trafficked via multivescicular bodies (MVBs), and degraded in lysosomes. Furthermore, we report that proteasome activity is required for the generation of the p116 cleaved ERBB2 in EE. Lastly, we also show that early autophagy is strongly inhibited upon GA treatment, therefore ruling out this catabolic pathway for the ERBB2 degradation in HSP90 inhibited cells. Altogether, our data support that HSP90 inhibition triggers multiple and coordinated events that orchestrate efficient ERBB2 down-regulation.

## RESULTS

### ERBB2 is preferentially internalized as full-length receptor

It has been reported that ERBB2 is internalized and degraded in lysosomes upon HSP90 inhibition [31, 33, 34]. To confirm this finding in our cell model system, we performed immunofluorescence studies to localize ERBB2 within the endosomal compartment. The data showed very poor or even absent co-localization between ERBB2 and lysosome LAMP-1 compartments upon GA treatment, as most of the internalized ERBB2 co-localized with TfR (Supplementary Figure S1). Moreover, since ERBB2 did not co-localize with the recycling endosome marker Rab11, we confirm our previous findings [31] suggesting that upon HSP90 inhibition the receptor does not follow its normal recycling fate. However, by using the modified immunoperoxidase DAB/AA method and Trastuzumab-HRP (TZ-HRP) as probe to detect ERBB2, we observed the localization of this receptor also in multivescicular bodies (MVBs) upon GA treatment (Supplementary Figure S2A and  S2B) [31].

These data can be either explained by a rapid ERBB2 degradation within lysosomes, or by an inefficient routing of the receptor to these compartments. However, as it has also been showed that ERBB2 undergoes a proteolytic cleavage upon GA treatment [2, 29, 30, 37], a third possible scenario could be that, upon ERBB2 cleavage, the receptor fragments follow a lysosome-independent degradation fate.

**Figure 1:**
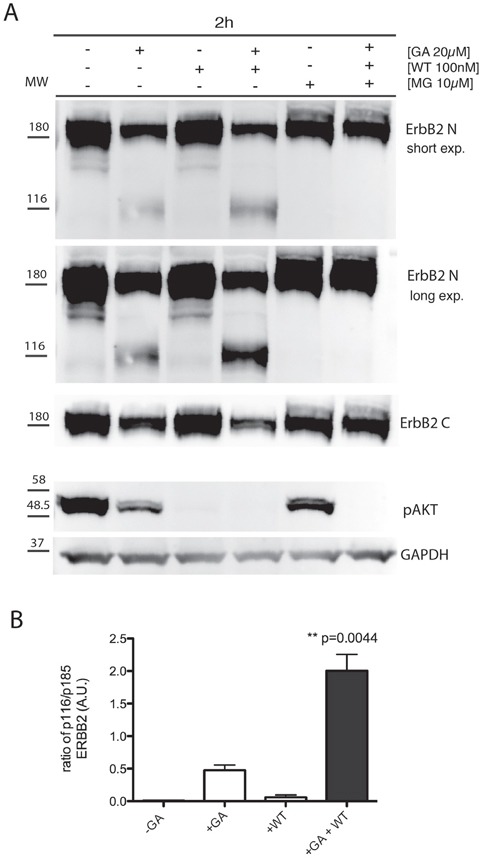
GA potentiates p116 ErbB2 cleavage which is enhanced when EE are inhibited with wortmannin **A.** SKBR3 cells were untreated or pre-treated with the PI3K inhibitor wortmannin (WT) (100nM), and/or the proteasome inhibitors MG132 (10μM) for 30 minutes at 37°C and GA was added for 1.5 hours at 37°C in the presence or absence of the inhibitors. Cell lysates were subjected to immunoblot analysis with antibodies to ERBB2 (Ab-20), ERBB2 (C-18) and p-AKT. GAPDH was used as loading control. Both a short and a long exposure (exp.) of the same blot are shown to better highlight the ERBB2 cleaved isoform, as indicated. The blot shown is representative of three-independent experiments. On the left side of each panel the migration of protein molecular mass standards expressed in kDa is shown. **B.** Histogram shows the ratio of p116 ERBB2 and p185 ERBB2. Bars represent the means ± SEM of three independent experiments (**p<0.05).

To discriminate among these scenarios, we first evaluated whether GA promotes ERBB2 cleavage in SKBR3 cells. By western blot analysis we observed that GA potentiates ERBB2 cleavage, leading to a 116 kDa trans-membrane N-terminal fragment, detectable with the Ab-20 antibody (Figure 1) (Table 1), recognizing the extracellular domain of ERBB2, but not with the C-18 antibody, recognizing the ERBB2 intracellular C-terminal domain (Figure 1).

**Table 1:** anti-ERBB2 antibodies used in this study

Antibody	Epitopes	Use in this study	Suppliers
9G6	N-terminal	IF	Santa Cruz Biotechnology
Ab-20	N-terminal	WB	Thermo
Trastuzumab	N-terminal	IP, IF, EM	Genentech-Roche
Ab-1	C-terminal	IF	Thermo
C-18	C-terminal	WB	Santa Cruz Biotechnology

**Figure 2:**
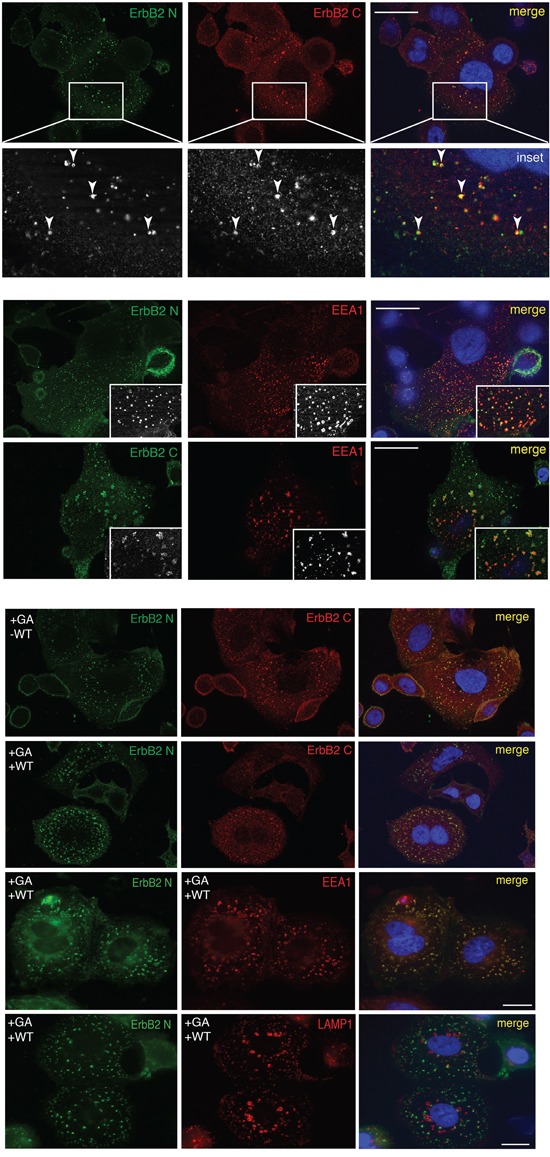
The full-lenght ERBB2 is trafficked to EEA1-positive compartments upon GA **A.** Internalized ERBB2 in GA-treated SKBR3 cells for 2 hours is detected by confocal immunofluorescence with antibodies against the extracellular (9G6, green signal) and intracellular (Ab-1, red signal) domains. Colocalization is shown as merged yellow signal (upper panel). The lower panel shows a magnified field of the areas boxed in the panels above. Arrows show colocalized signals. SKBR3 cells treated with GA for 2 hours and ERBB2 localization (9G6 and Ab-1, green signal) with endogenous EEA1 (red signal) is shown. Insets show signal colocalization at a higher magnification. Scale bars: 20μM. **B.** Effects of GA in combination with WT on ERBB2 localization detected by confocal immunofluorescence. Images show intracellular and plasma membrane localization of ERBB2 detected with anti-ERBB2 (green signal, 9G6) and with anti-ERBB2 antibody (red signal, Ab-1). In addition, co-localization of internalized ERBB2 (9G6) with the EE marker EEA1 and the lysosomal marker LAMP1 in GA+WT treated cells is shown. Colocalization is shown as yellow signal in the merged images. Size bar: 20μM.

We next investigated whether ERBB2 cleavage events are necessary for the receptor internalization, and performed a double immunofluorescence analysis in GA-treated and untreated cells, using 9G6 and Ab-1 antibodies. Both antibodies co-localized within EEA1-labeled EE (Figure 2A), suggesting that ERBB2 is preferentially internalized and routed to EE as a full-length receptor. Of note, the EEA1 compartment in GA treated cells displayed a different morphology compared to untreated cells, as in the former EE appeared irregular in shape, in accordance to the ultrastructure alterations of EE and MVBs described in GA treated cells [31, 34], and significantly increased in size (0.78μm vs 0.47μm in controls), whereas untreated cells displayed a rather round shape, with a peripheral labeling (Supplementary Figure S3).

### GA-induced ERBB2 cleavage occurs in early endosomes

As ERBB2 reaches EE uncleaved, upon GA treatment, we wanted to further investigate whether the GA-induced ERBB2 cleavage occurred within the altered EE compartment, or at later compartments. To this aim we took advantage of wortmannin (WT), a potent inhibitor of PI3K activity, which is known to dissociate EEA1 from EE, thus impairing fusion/trafficking and the maturation events involving these compartments [40, 41]. SKBR3 cells were pre-treated with WT (100nM), for 30 minutes at 37°C and then treated with GA. The effectiveness of the WT treatment was confirmed by the disappearance of the phospho-AKT (p-AKT) signal, the major downstream signaling readout of ERBB2, in immunoblot analysis, whose phosphorylation depends on PI3K activity, in all the conditions of WT treatment, irrespective of ERBB2 cleavage (Figure 1).

By immunofluorescence analysis, we localized ERBB2 using both the 9G6 antibody, directed to the extracellular domain, and the Ab-1 antibody, to the intracellular domain (Table 1). In GA treated cells we calculated the percentage of colocalization of internalized ERBB2. The overlapping signal of the two antibodies was 80%, meaning that the majority of ERBB2 is internalized as full-length p185 isoform (Supplementary Figure S4). In cells treated with GA and WT, both ERBB2 isoforms p116 (9G6 staining alone), and p185 (9G6 and Ab-1 staining overlapping) were present within EEA1-enlarged compartments, whereas only a faint plasma membrane labeling was detected (Figure 2B). Supplementary Figure S5 shows that the antibody 9G6 specifically recognized the p116 ERBB2 isoform under the experimental conditions used to perform immunofluorescence analysis. Notably, no ERBB2 staining co-localized with LAMP-1 in cells treated with GA and WT (Figure 2B), at variance to the mild localization observed in cells treated with GA in the absence of WT (Supplementary Figure S1), suggesting that ERBB2 trafficking was blocked in EE. These results support the hypothesis that ERBB2 cleavage occurs in EE. To further support this hypothesis, we performed western blot analysis of cells treated or untreated with GA, in the presence or in the absence of WT. By revealing ERBB2 using either the Ab-20, or the C-18 antibodies, we observed no differences in the ERBB2 levels in GA untreated cells, both in the presence or in the absence of WT (Figure 1), as expected. On the contrary, the presence of GA promoted the formation of the p116-ERBB2 that was higher in the presence of both GA and WT. These data suggest that impairment of the EE maturation into late endosomes, by WT, allows accumulation of the p116 fragment in the EE compartment (Figure 1 and 2B), supporting a model in which GA promotes ERBB2 internalization into EE, followed by cleavage into a p116 fragment.

### Proteasome activity is required for ERBB2 cleavage in EE

**Figure 3:**
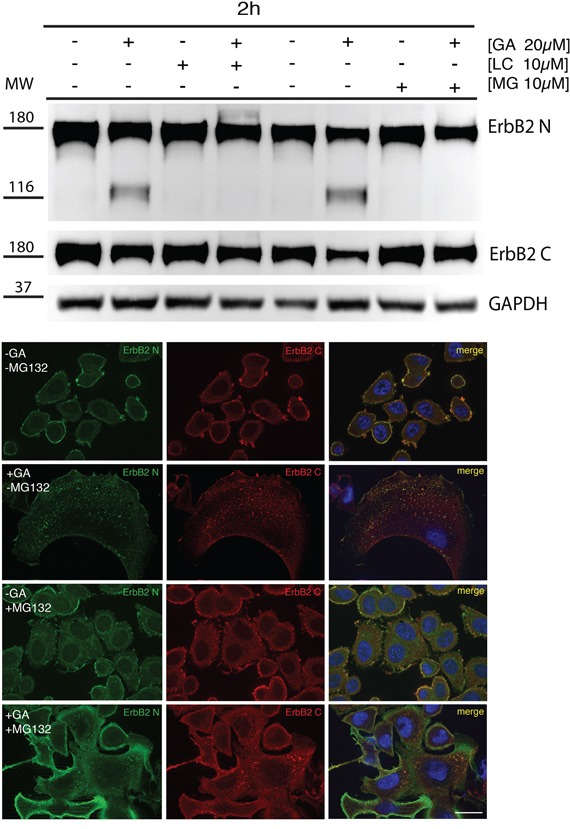
Proteasome is responsible for the generation of p116-ERBB2 under GA **A.** SKBR3 cells were pre-treated with the proteasome inhibitors MG132 (10μM) and lactacystin (LC) (10μM) for 30 minutes at 37°C and GA was added for 1.5 hours at 37°C in the presence of the inhibitors. Note that p116-ERBB2 is detectable only after GA treatment, and that ERBB2 cleavage is completely prevented when the proteasome activity is inhibited (Ab-20, upper panel). In the middle panel, p185-ERBB2 was detected with C-18 antibody. Note that, in both GA+LC and GA+MG132 treated cells ERBB2 degradation of the p185 isoform, as detected by C-18 Ab, is inhibited if compared to GA treated cells. GAPDH was used as loading control (lower panel). The blot shown is representative of three-independent experiments and the left four samples belong to a different experiment respect to the right four samples. On the left side of each panel the migration of protein molecular mass standards expressed in kDa is shown. **B.** ERBB2 localization upon proteasomal inhibition with MG132. Representative confocal images of ERBB2 intracellular localization obtained from untreated or GA-treated SKBR3 cells in the presence or absence of 10μM MG132. The green signal shows ERBB2 extracellular domain (9G6), the red signal shows ERBB2 intracellular domain (Ab-1). Colocalization is shown as yellow signal. Full-length ERBB2 (yellow signal) is detectable at the intracellular level in both GA-treated and co-treated with MG132. Notably, enlarged ERBB2-positive endosomal structures and plasma membrane staining are visible in GA+MG132 co-treated cells. Images are representative of three independent experiments. Scale bars: 20 μm.

**Figure 4:**
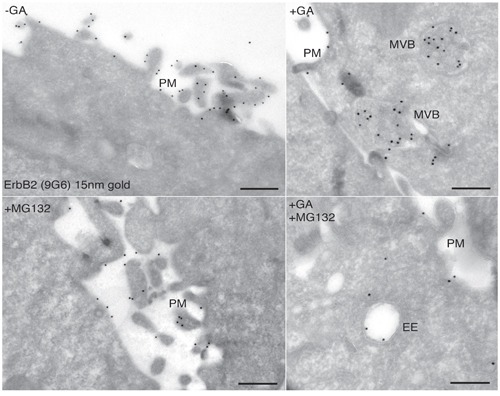
Ultrastructural localization of ERBB2 in SKBR3 cells treated with GA and proteasome inhibitors Cryosections of SKBR3 cells were labeled with antibodies against ERBB2 (9G6), followed by protein A-coated (15nm) colloidal gold (black dots). The labeling shows that ERBB2 localized to the highly ruffled plasma membrane in untreated cells **A.**, and mainly in multivescicular bodies (MVBs) in GA-treated cells **B.** Cells treated with 10μM MG132 show plasma membrane localization of ERBB2, as in untreated cells **C.** Cells co-treated with GA and MG132 show intracellular localization of ERBB2 in morphologically resembling early/recycling endosomes (D).

Contrasting results have been reported with respect to proteasome requirement for ERBB2 cleavage, endocytosis and degradation under GA inhibition [36, 38]. To test whether the proteasome is involved in the generation of the p116-ERBB2 isoform, we performed experiments in the presence of two proteasome inhibitors, i.e. MG-132 and lactacystin (LC). MG132 is a potent reversible inhibitor of chymotrypsin-like (CT-L) activity and of the cysteine protease calpain-1, whereas LC is an irreversible and efficient inhibitor of proteasome CT-L activity, but has no activity on cysteine and serine proteases. SKBR3 cells pre-incubated with 10μM LC, or 10μM MG132, for 30 minutes at 37°C, were treated with 20μM GA in the presence of LC or MG132, for further 2 hours at 37°C. Under these conditions, we observed that proteasome inhibition efficiently prevents the GA-dependent ERBB2 cleavage (Figure 3A). Double immunofluorescence analysis, using the 9G6 and the Ab-1 antibodies, showed that in the presence of GA and MG132 the uncleaved ERBB2 is mainly localized in enlarged intracellular compartments, and on the plasma membrane (Figure 3B and Supplementary Figure S6A). However, if GA treatment was combined with MG132 and WT, the plasma membrane labeling disappears and the uncleaved ERBB2 remains localized in even larger intracellular compartments (Supplementary Figure S6B), supporting the hypothesis for a proteasome role in ERBB2 cleavage in the EE. To further characterize these compartments we performed an immunoelectron microscopy analysis on cryosections, and found that while in GA treated cells ERBB2 localizes within MVBs, in the presence of GA and MG132, the receptor is localized in EE/recycling compartments, and at the cell surface (Figure 4).

Collectively, these data strongly suggest that proteasome activity is required for ERBB2 cleavage within EE, and suggest that this event is necessary for the further trafficking to late/lysosomal compartments. Of note, when LC was used instead of MG132 to inhibit the proteasome activity, we obtained similar results (not shown).

MG132 has an inhibitory activity on calpain-1, and calpain-1 associates to HSP90 and appears to be implicated in constitutive ERBB2 digestion in *in vitro* experiments [42, 43]. Therefore, we wanted to rule-out the role of this non-lysosomal cysteine protease in the GA-dependent generation of p116-ERBB2. Taking advantage of the specific calpain inhibitor-1 (ALLN), which reversibly blocks calpain-1 activity at concentrations ranging between 1-2μM. We pre-treated SKBr3 cells with 2μM ALLN for 30 minutes at 37°C and subsequently added 20μM GA for 2 hours at 37°C in the presence of the inhibitor, and analyzed the cells by western blot and immunofluorescence. The results show that ALLN did not prevent either ERBB2 cleavage or endocytosis in the presence of GA (Supplementary Figure S7). Accordingly, activation of calpain-1 with 1μM Ca^2+^ ionophore did not induce fragmentation of ERBB2 (Supplementary Figure S7). These results rule out the involvement of calpain-1 in GA-dependent ERBB2 cleavage and endocytosis and suggest that ERBB2 cleavage depends on the activity of the proteasome.

### ERBB2 is polyubiquitinated in GA-treated cells

**Figure 5:**
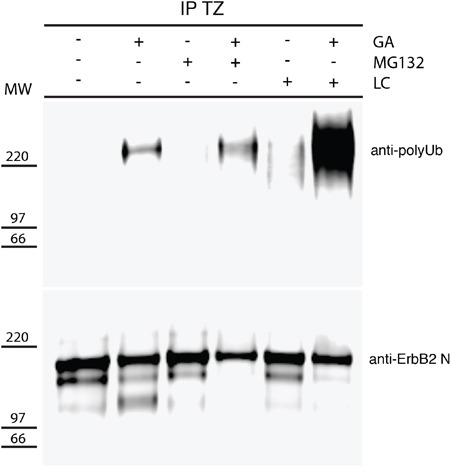
GA induces polyubiquitination of p185-ERBB2 SKBR3 cells were pre-treated with the proteasome inhibitors MG132 (10μM) and lactacystin (LC) (10μM) for 30 minutes at 37°C and GA was added for 1.5 hours at 37°C in the presence or absence of the inhibitors. Cells were then lysed as described under the material and method section. The cell lysates were immunoprecipitated with trastuzumab (TZ) in order to detect ERBB2. Immunoprecipitates were resolved by SDS/PAGE and immunoblotted with an anti-polyubiquitin (upper panel), and an anti-ERBB2 (Ab-20) antibody (lower panel) are shown. On the left side of the panels the migration of protein molecular mass standards expressed in kDa is shown.

As proteasome inhibitors impaired the formation of p116-ERBB2, we sought to confirm in our model system the reported GA-dependent polyubiquitination of ERBB2 [23]. We immunoprecipitated ERBB2 from GA treated and control untreated cells, using TZ, and revealed polyubiquitination by western blot analysis, using a specific antibody recognizing polyubiquitin chains. As expected [23, 44], HSP90 inhibition promotes the polyubiquitination of ERBB2 (Figure 5), but not of TfR immunoprecipitated with a specific antibody, used as a negative control (Supplementary Figure S8). In particular, in the TZ immunoprecipitates, the anti-polyubiquitin antibody recognized a band migrating slower than 185kDa, confirming the polyubiquitination of the full-length ERBB2 form (Figure 5). However, challenging the same blot with Ab-20, revealed only the non-ubiquitinated ERBB2 isoforms (Figure 5), suggesting that only a minor amount of ERBB2 is polyubiquitinated and likely rapidly degraded. In addition, to determine whether proteasome inhibition affects the levels of polyubiquitinated ERBB2, we immunoprecipitated ERBB2 from cells co-treated with GA and MG-132 and with GA and LC, using TZ, and revealed the levels of polyubiquitination by western blot analysis (Figure 5), and found that MG-132 and LC increased the abundance of polyubiquitinated ERBB2 in the presence of GA, with LC having a more robust effect than MG-132. These results confirm that full-length ERBB2 is polyubiquitinated under GA and that proteasome inhibition induces accumulation of polyubiquitinated ERBB2.

### Degradation of the p116-ERBB2 fragment occurs in the endosome/lysosome compartments

**Figure 6:**
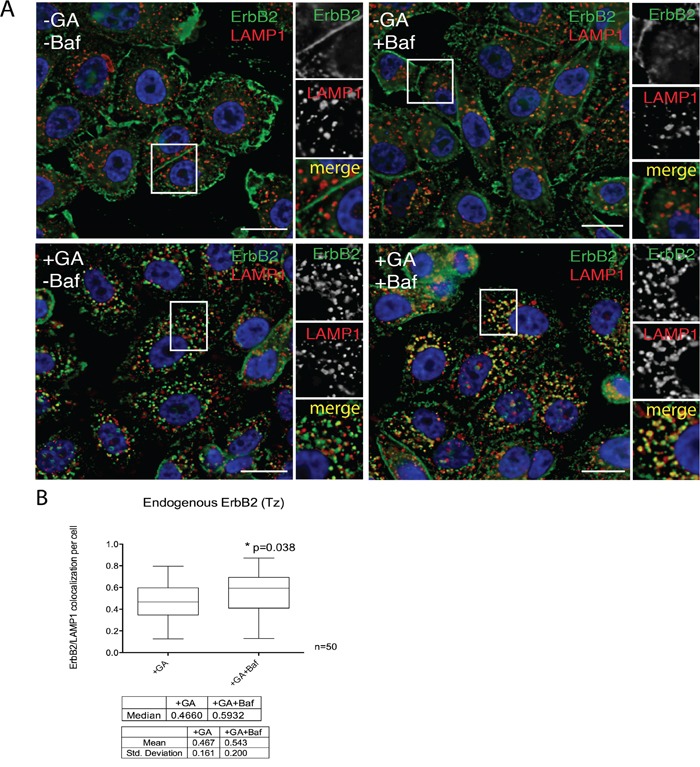
The N-terminal ERBB2 fragment is readily delivered to lysosomes **A.** SKBR3 cells were pre-incubated with 200nM BafilomycinA1 (Baf) for 30 minutes at 37°C and then with 20μM GA, Baf and GA+Baf for 1.5 hours at 37°C. Cells were fixed and labelled for ERBB2 (9G6, green signal) and the lysosomal marker LAMP1 (red signal). Representative confocal microscopy images are shown. Note that in the case of GA-treated cells, only few ERBB2 is found inside lysosomes, whereas the colocalization is greatly increased when Baf was added in co-treatment with GA. Insets on the right side of each image show at higher magnification the boxed area. Size bars: 10μM. **B.** Pixel quantification of ERBB2-LAMP1 colocalization per cell (n=3, 50 cells analyzed each experiment) in GA versus GA+Baf treated cells is depicted as box plot. The bottom and the top of each box are the first and third quartile, while the line inside the box represents the median (second quartile). The ends of the whiskers represent the minimum and the maximum data value. A significant increase (p=0.038) of ERBB2 colocalization with LAMP1 is shown when lysosomal activity was inhibited by Baf. Images and quantification are representative of three independent experiments.

Our results indicate so far that, upon GA treatment, ERBB2 is cleaved in EE giving origin to a p116 protein, in a proteasome-dependent manner. We next asked whether the degradation of p116-ERBB2 occurs in the late endosome/lysosome, or in EE compartments. To answer this question, we localized ERBB2 by immunofluorescence in GA treated cells, in the presence or in the absence of the lysosomal inhibitor bafilomycin A1 (Baf), a specific inhibitor of the vacuolar H+ ATPase (V-ATPase). The co-localization between endogenous ERBB2 and LAMP-1 (Figure 6A) increased in the presence of Baf, compared to control cells (Figure 6B). These results support the conclusion that ERBB2 cleaved in EE is eventually rapidly degraded in lysosomes, so that it becomes promptly detectable only when their activity is inhibited.

**Figure 7:**
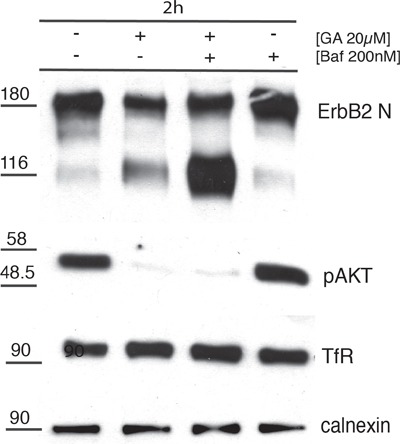
p116-ERBB2 isoform is signaling impaired and degraded in lysosomes SKBR3 cells were untreated or treated with 20μM GA, 200nM BafilomycinA1 (Baf) alone or in combination for 2 hours at 37°C. The total p185-ERBB2, the GA-induced p116-ERBB2, p-AKT and TfR protein levels were assessed by Western blot using Abs recognizing the extracellular domain of ERBB2 (Ab-20), p-AKT and TfR. Calnexin was used as loading control. On the left side of the blot the migration of protein molecular mass standards expressed in kDa is shown.

These results were confirmed by biochemical analysis. SKBR3 cells were incubated with either one of two lysosome inhibitors, i.e. 200nM Baf (Figure 7), or 200nM cloroquine (CQ)(Supplementary Figure S9), in the presence or in the absence of GA. Indeed, the p116 fragment was less abundant (Figure 7) in cells treated with GA, whereas it was greatly accumulated when the degradative activity of lysosomes was inhibited by Baf (Figure 7). Furthermore, the levels of p-AKT, were almost abolished upon GA treatment, either in the presence or in the absence of Baf (Figure 7). This further supports the conclusion that the cleavage leading to the formation of the signaling-impaired p116-ERBB2 fragment, lacking the carboxy-terminal kinase domain, occurred in EE, before the receptor reached the degradation sites. This conclusion was also in agreement with the observation that Baf failed to inhibit ERBB2 cleavage (Figure 7).

We have previously reported that GA treatment re-routes recycling cargoes (i.e. ERBB2 and TfR) from early/recycling compartments to late MVBs [31]. These events involve the reshaping of endocytic compartments. We now find that ERBB2 trafficking to late endosomes/lysosome compartments upon GA treatment follows a proteasome cleavage of the receptor in a 116 fragment, in the EE. To better characterize the endocytic compartments involved ERBB2 trafficking in cells treated by GA in the presence of Baf, we took advantage of the modified immunoperoxidase DAB/AA method we reported earlier [45], and used TZ-HRP to track ERBB2. Briefly, SKBR3 cells were pre-treated with 20μM GA for 20 min at 37°C, transferred on ice, and labeled for 15 minutes with TZ-HRP. After washing out the excess of TZ-HRP, cells were incubated for 2 hours at 37°C with or without GA, in the presence or in the absence of Baf. By morphology, we identified four classes of ERBB2-positive endocytic structures in GA and GA+Baf treated cells: endosomes (∼250nm), small multivesicular bodies (sMVBs) (∼100nm, 1-2 intraluminal vesicles), MVBs (∼300nm, >10 intraluminal vesicles), and an unconventional compartment that we named bafilomycin-derived vesicles (BDVs, ∼300nm) (Supplementary Figure S2A). By morphometry, we quantitated the surface occupancy of each of these compartments in the different treatment conditions (Supplementary Figure S2B). The data confirmed the ERBB2 routing to MVBs, as we previously described [31], and revealed defects in MVB maturation/fusion in the presence of GA+Baf, represented by MVB smaller in size and often clustered in discrete regions of the cytoplasm (small MVBs) [46], and the appearance of BDVs, electron-lucent endosomal structures containing a weak TZ-HRP labeling, that represented the most frequent phenotype.

Since TZ has been shown to modulate intracellular signaling pathways [3, 47], we wanted to rule out the possibility that the TZ used to track ERBB2 in our experiments could interfere with the GA-induced ERBB2 cleavage, trafficking, degradation and downstream signaling. By western blot analysis we found that treatment with TZ alone does not generate the 116kD cleaved ERBB2 fragment, and does not interfere with the cleavage induced by GA. Accordingly, lack of p-AKT phosphorylation was also observed in GA+TZ treated cells (Supplementary Figure S10A). Moreover, we checked whether TZ was interfering with the GA-induced ERBB2 trafficking, by immunofluorescence. The antibody TZ-Alexa488 was allowed to internalize for 2 hours at 37°C in cells treated or untreated with GA, in the presence or the absence of Baf. Co-localization studies with LAMP-1 revealed no differences in ERBB2 trafficking to lysosomes using TZ-Alexa488 compared with endogenous ERBB2 trafficking, supporting that, in the presence of GA, TZ is affecting neither ERBB2 cleavage nor its trafficking (Supplementary Figure S10B- S10C).

### Autophagy is not a relevant mechanism for GA-mediated ERBB2 down-regulation

**Figure 8:**
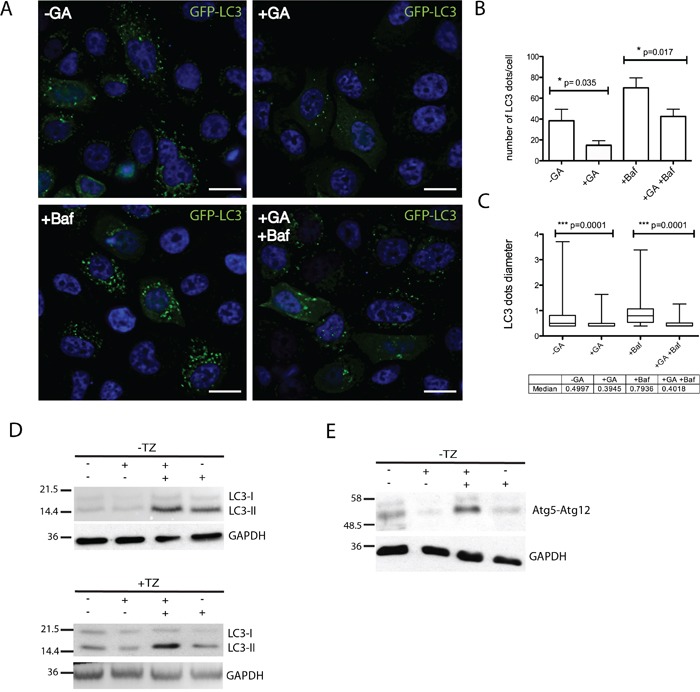
GA inhibits early autophagy **A.** SKBR3 transiently transfected with GFP-LC3 for 18 hours and then treated with GA, BafilomycinA1 (Baf) and GA+Baf for 2 hours at 37°C (green signal, GFP-LC3; blue signal, DAPI). Representative images are shown from two independent experiments. Scale bars: 10μM. Note that in GA-treated cells few GFP-LC3 dots are visible if compared to untreated and Baf-treated cells. **B.** Quantification of the average number of LC3 dots in cells (n=50) shows that GA significantly decreases LC3-positive autophagosomes (p=0.035) compared to untreated cells. Accordingly, when Baf was added to inhibit autophagosome/lysosomal fusion, GA treatment decreases the number of LC3-positive autophagosomes (p=0.017). **C.** Quantification of the average size of LC3 dots in SKBR3 cells (n=3, 50 cells analyzed each experiment). GA significantly decreases LC3-positive autophagosomes size (p=0.001) either alone or in co-treatment with Baf. The bottom and the top of each box are the first and third quartile, while the line inside the box represents the median (second quartile). The ends of the wiskers represent the minimum and the maximum data value. **D.** Autophagic flux was assessed by western blot analysis in SKBR3 cells treated with GA, Baf and GA+Baf for 2 hours at 37°C in the absence or presence of TZ. Accordingly with data presented in (A), LC3-II levels were decreased in the presence of GA. The level of LC3-II is increased in the presence of the lysosomal inhibitor Baf because the transit of LC3-II through the autophagic pathway is blocked, but is decreased in GA+Baf treated cells, indicating inhibition of early autophagy. TZ does not influence the inhibition of autophagy mediated by GA. **E.** Protein levels of the early autophagy marker atg5-atg12 complex is assessed by western blot analysis in SKBR3 cells treated with GA, Baf and GA+Baf for 2 hours at 37°C. It is shown that GA reduces the level of this complex. GAPDH was used as loading control for all the experiments. On the left side of each panel the migration of protein molecular mass standards expressed in kDa is shown.

Recent work demonstrated that certain conditions that promote ERBB2 polyubiquitination may lead to the receptor degradation by autophagy [6]. Therefore, we were prompted to evaluate whether autophagy could be a possible degradation modality in GA treated cells. By measuring LC3-II levels as a functional reporter of autophagy [48, 49], we first, transiently transfected SKBR3 with GFP-LC3 for 18 hours and scored by immunofluorescence the number of LC3 dots under different treatment conditions. We found that GA treatment reduced the number of LC3-II dots within cells (Figure 8A-8C), suggesting that HSP90 inhibition suppresses autophagy in contrast to previous studies in different experimental settings [50, 51]. To assess whether the LC3-II reduction resulted from either decreased autophagosomes synthesis, or from an increased degradation, we evaluated the autophagic flux in the presence of Baf, that is known to block autophagosome degradation [49, 52]. SKBR3 cells treated or untreated with GA, in the presence or the absence of Baf, were analyzed by western blot for LC3I-II levels (Figure 8D). LC3-II levels were lower in the conditions without Baf, compared to treatments in the presence of the inhibitor, confirming that GA reduced LC3-II levels by interfering with autophagosome synthesis. As a second approach, we took advantage of the observation that the activity of the conserved Atg12-Atg5-Atg16 complex is essential for initial autophagosome biogenesis [53]. Therefore, by western blotting we analyzed the levels of Atg5-Atg12 complex in cells treated or not with GA, in the presence or the absence of Baf (Figure 8C). As expected, GA down-regulates the Atg5-Agt12 complex, further confirming its role in impairing autophagosome biogenesis. Of note, we could not detect ERBB2-positive autophagic structures in the EM analysis in cells treated with GA, irrespective of the presence or not of Baf (Supplementary Figure S2A). Collectively, these results suggest that early autophagy is strongly inhibited in GA-treated cells, excluding this catabolic pathway for ERBB2 degradation.

## DISCUSSION

Overexpression of ERBB2 in breast cancers correlates with a poor prognosis due to enhanced metastatic potential and resistance to chemotherapy. For these reasons, many efforts have been spent in the development of therapeutic antibodies (i.e. TZ) [54, 55] and HSP90 inhibitors that have shown to be able to stimulate ERBB2 endocytosis and termination of downstream signaling. Since the discovery of the natural product GA in 1970, the portfolio of HSP90 inhibitors has been greatly expanded and includes multiple drug classes, with different modes of action, that are undergoing clinical evaluation [14, 17]. ERBB2 is “addicted” to HSP90 and is very sensitive to HSP90 inhibition in pre-clinical models. In the clinic, the efficacy of this class of therapeutic agents has been relatively limited, with promising responses mainly observed in ERBB2^+^ breast and lung cancers. Notably, the GA analog 17-AAG combined with TZ showed increased clinical efficacy in ERBB2^+^ breast cancer patients that become resistant to TZ, highlighting HSP90 inhibitors as effective agents to overcome or prevent drug resistance [56]. At the present, the knowledge of the basic cellular and molecular mechanisms underlying ERBB2 down-regulation under HSP90 inhibition is still not completely clear.

In particular, endocytic down-regulation is a recurrent theme in ERBB cancer biology. Indeed, ERBB2 has been considered either to be endocytosis-resistant, or a fast recycling receptor confined to the plasma membrane [11, 34].

The objectives of this study were to determine the sequence of events leading to ERBB2 degradation upon HSP90 inhibition, to understand the mechanism of action of potentially useful anti-cancer drugs, and to better understand the biology of the role of HSP90 in the homeostasis of ERBB2.

Our data suggest a model for ERBB2 degradation induced by GA, which can be described as a four steps process: first, ERBB2 polyubiquitation of the full length p185-ERBB2 cytoplasmic tail; second, internalization and trafficking of the polyubiquitinated receptor to EE; third, the generation of a p116-ERBB2 cleaved fragment, by proteasome removal of the ubiquitinated cytoplasmic tail from the p185-ERBB2; and fourth, trafficking to late endosomes/lysosomes and rapid degradation of the p116-ERBB2 fragment. In addition, we suggest that the p116/p185 ERBB2 ratio could be of help in the assessment of response to GA derivatives in tumor-derived organoids.

Some of our findings confirm previous studies, in particular here we show that GA promotes the generation of a p116-ERBB2 signaling impaired fragment in SKBR3 cells, which overexpress the endogenous ERBB2 due to *ERBB2* gene amplification. Previous findings reported the generation of a 135kDa fragment upon GA treatment [29], this discrepancy in fragments mass is likely due to the use of an ERBB2/ERBB1 chimeric construct expressed in NIH3T3 mouse fibroblasts [29]. In addition, our finding of ERBB2 polyubiquitination and cleavage induced by GA treatment confirm previous studies [23, 29, 39].

However, here we define for the first time that the ERBB2 polyubiquitination occurs on the full-length p185 receptor, and it is a necessary event for the cleavage of ERBB2 cytoplasmic tail, due to proteasome activity in EE. These findings reconcile two not mutually exclusive hypotheses on the role of polyubiquitination in GA-mediated ERBB2 down-regulation. In the first, polyubiquitin chains have been suggested to serve as endocytic sorting signal, triggering internalization of full-length ERBB2 from the plasma membrane, as suggested by the observation that a tetra-Ub-containing fusion protein, ERBB2-Ub_4_, is constitutively endocytosed, and not cleaved in the absence of the GA analog 17-AAG [39]. In the second hypothesis, polyubiquitination has been proposed to represent the molecular signal triggering the intracellular cleavage, necessary to sort ERBB2 to EE and then multivescicular bodies (MVBs), en route to lysosomes [10].

In particular, we provide evidences that cleavage of the polyubiquitinated p185-ERBB2 occurs in EE, before final degradation of the p116-ERBB2 in the late endosome/lysosome compartments. In agreement with the original observations by Lerdrup and by Pedersen [36, 38], we found p185-ERBB2 associated to EEA1-positive endosomes, when using antibodies recognizing both the extracellular and intracellular regions of ERBB2 in GA treated cells, strongly suggesting that ERBB2 is not cleaved before endocytosis. In addition, impairing EE fusion/trafficking with the PI3K inhibitor wortmannin (WT) [41] in GA treated cells, leads to an increase in the p116-ERBB2 isoform within enlarged EEA1-endosomes, and not in LAMP1 lysosomes. It should be noticed that both GA analogues and PI3K inhibitors are either under development in clinical trials, or already approved. Based on our data, it is possible to speculate that the combined use of these drugs may turn out to be effective in reducing breast cancer cells proliferation/survival in vivo.

Of note, the ERBB2 positive EE observed in GA treated cells were morphologically altered, as we have previously demonstrated by ultrastructural studies [31].

A further unclear aspect of the GA dependent events leading to ERBB2 degradation was the precise role of the proteasome for ERBB2 down-regulation, which has long been a matter of debate [29, 36, 38]. In this study, we show by biochemical analysis that proteasome inhibition by MG132, in the presence of GA, dramatically impairs the generation of p116-ERBB2 isoform, even in cells co-treated with WT, leading to accumulation of polyubiquitinated p185-ERBB2. This finding suggests that ERBB2 cleavage in the EE and the subsequent p116-ERBB2 sorting to late endosomes/lysosomes is dependent upon proteasome activity. Of note, under GA and proteasome inhibition, we observe an increase of ERBB2 at the plasma membrane in a large fraction of cells, possibly because of the impaired trafficking of the polyubiquitinated p185-ERBB2 to the receptor-saturated EE. By immunoelectron microscopy we find the receptor associated to intraluminal vesicles of MVBs upon GA treatment. However, when cells are co-treated with the proteasome inhibitor MG132, ERBB2 localizes to compartments with EE morphology, containing few or no internal vesicles, and with the labeling restricted to the outer limiting membrane, a typical feature of recycling receptors [57]. These observations provide a likely scenario for the sequence of events preluding to the ERBB2 missorting from recycling, to degradation in late endosomes/lysosomes, that we have previously described [31].

Therefore, we believe that the overall GA-induced p116 cleavage and subsequent EE/LE trafficking, but not its internalization, depends on proteasome activity.

MG132 was reported to also inhibit calpain-1. The involvement of calpain-1 in regulation of TZ sensitivity and survival pathway by regulating cleavage and activity of ERBB2 in breast cancers has been recently investigated [42]. In addition, calpain-1 associates with HSP90, in which the chaperone appears to restrain its proteolytic activity [43]. Based on these findings, we reasoned that GA might influence the protease activity of calpain-1 in SKBR3 cells. However, we found that specific inhibition of calpain-1 with the calpain-1 inhibitor (ALLN) had no effect on p116-ERBB2 isoform generation and ERBB2 internalization in GA-treated cells. Therefore, we concluded that the observed effects of MG132 on ERBB2 cleavage were not dependent from calpain-1. Lastly, we asked whether the degradation of the ERBB2 p116 fragment occurred in the lysosomal compartments. Our biochemical and immunofluorescence data showed that the p116 isoform was greatly accumulated in lysosomes when their activity was inhibited with bafilomycin A1, confirming that its final fate is the lysosomal degradation.

In parallel to endocytosis, autophagy represents another major pathway for protein degradation sharing with endocytosis part of the molecular machinery [58]. In cancer cells, autophagy has dual roles, acting as both a tumor suppressor by preventing the accumulation of damaged proteins and organelles and as a mechanism of cell survival that can promote the growth of established tumors [59]. Recently, a cross-talk between polyubiquitination of surface receptors and autophagy-mediated degradation has been demonstrated [60]. In breast cancer cells, when the p130Cas adaptor protein is depleted, ERBB2 becomes ubiquitinated by c-cbl and degraded via autophagy [6]. In our study, we found that GA strongly impaired early autophagy, as shown by the decrease of LC3-II and Atg5-12 complex levels, highlighting GA as a potent early autophagy inhibitor in breast cancer SKBR3 cells. These results indicate that HSP90 regulates early steps of autophagy as part of its anti-cancer activity, but it is likely not involved in degradation of polyubiquitinated ERBB2.

In conclusion, our study shows that as a result of the coordinated activities of ubiquitin/proteasome and endocytic pathways, the full-length ERBB2 is polyubiquitinated, internalized and sorted to EE, cleaved into a p116 isoform, en route to degradation in lysosomes. GA treatment potentiates these events and in addition acts as potent early autophagy inhibitor in our experimental settings. Together, our findings provide novel insights into the role of HSP90 in ERBB2 processing, and of the molecular mechanisms underlying HSP90 inhibition in SKBR3 breast cancer cells.

## MATERIALS AND METHODS

### Cell culture

Human breast cancer (SKBR3) overexpressing ERBB2 were obtained from ATCC. Cells were grown in Dulbecco's modified Eagle's medium (DMEM; Gibco, Carlsbad, CA, USA) supplemented with 10% heat inactivated Fetal Bovine Serum and 2mM glutamine (Gibco, Carlsbad, CA, USA) and maintained in 5% CO2. Confluent cells were split with Trypsin-EDTA (Gibco, Carlsbad, CA, USA) and passaged at an appropriate density. SKBR3 cells were transiently transfected with Lipofectamine 2000 (Invitrogen, Carlsbad, CA, USA) and examined 18 hours post-transfection according to instructions.

### Antibodies and reagents

Antibodies against ERBB2 are listed in Table 1 along with epitope specificity, applications and suppliers. Of note, Trastuzumab (Trastuzumab®, Genentech and Roche, South San Francisco, USA) was from the week residues of the pharmacy of IST (Istituto Nazionale Tumori, Genova, Italy) kindly donated to Carlo Tacchetti for research purposes, at a concentration of 21mg/ml.

Rabbit polyclonal anti-EEA1 (H-300) was from Santa Cruz. Mouse monoclonal anti-LAMP1 (H4A4) was from Hybridoma Bank (Iowa City, Iowa, USA). Rabbit polyclonal anti-LAMP1 (L1418) was from Sigma-Aldrich. Mouse anti-human Transferrin receptor (clone H68.4) was from Invitrogen. Anti-LC3 (clone 5F10) antibody was from Nanotools. Rabbit polyclonal anti-ATG5 antibody was purchased from Novus Biological. Antibodies to pan-AKT and p-AKT were purchased from Cell Signaling. Anti-polyubiquitin antibody was purchased from Millipore (clone FK1). Trastuzumab® conjugation with HRP were made using Lightning-Link TM technology (Innova Bioscience, Cambridge, UK) according to manufacter's instructions. Trastuzumab conjugation with Alexa488 and Alexa555 dye was made with Alexa Fluor conjugation kit (A10235, Invitrogen, Carlsbad, CA, USA). Geldanamycin, BafilomycinA1, ascorbic acid and DAB (3,3-Diaminobenzidine tetrahydrochloride) tablets (D5905) were from Sigma-Aldrich (St. Louis, MO, USA). Calpain-1 inhibitor (A6185, Sigma-Aldrich) was a gift from M. Averna (Dipartimento di Medicina Sperimentale, Biochemistry section, Università di Genova). Proteasome inhibitors MG-132 (474790) and Lactacystin (416100) were purchased from (MERK-Millipore, Darmstadt, Germany). Protein A-gold (10-15nm) was purchased from Utrecht University, Utrecht, Netherlands.

### Electron microscopy

#### Trastuzumab-HRP internalization assay

SKBR3 cells, cultured on 2-well Lab-Tek chamber Slide w/cover permanox (177429, Thermo Fisher Scientific, 75 Panorama Creek Drive, Rochester, NY) were washed in CO2-independent medium (Gibco, Cat. Number 18045070, Carlsbad, CA, USA), incubated in CO2-independent medium containing 10 μg/ml TZ-HRP on ice for 30 minutes in the presence or in the absence of 20μM GA and 200nM BafilomycinA1. After extensive wash with cold CO2-independent medium to remove unbound TZ-HRP, cells were shifted at 37°C in CO2-independent medium for 2 hours at 37C, in the presence/absence of the drug/s. To stop the internalization, cells were placed on ice in pre-chilled CO2-independent medium, incubated for 20 min at 4°C in freshly prepared DAB buffer (1 mg/ml DAB and 0.012% H_2_O_2_) with or without 50 mM ascorbic acid (AA) in PBS and fixed with 2.5% glutaraldehyde (Electron Microscopy Science, Hatfield, PA, USA) for 1 h. The cells were subsequently processed for standard EM. Cells were embedded in Epon epoxy resin (Poly/Bed® 812, Polyscience, Valley Road Warrington, PA, USA) and cut parallel to the culture substratum. Electron micrographs were taken at CM10 TEM (Philips, The Netherlands) and analyzed with iTEM software (Olympus-SYS, Münster, Germany). Quantification of TZ-HRP internalization (±GA, ± BafilomycinA1 or both) by EM is represented as histogram in which the ratio of the average area occupied by each type of ERBB2-positive endocytic structure with the average area of each image was calculated (mean±SEM). We defined four classes of ERBB2-positive structures in GA+BafilomycinA1 co-treated cells by morphological criteria: endosomes (∼240nm), small multivesicular bodies (sMVBs) (∼100nm, 1-2 intraluminal vesicles), MVBs (∼310nm, >10 intraluminal vesicles), bafilomycin-derived vescicles (BDVs) (electron-lucent endosomes, ∼300nm). We analyzed 30 images for each treatment (magnification of 19500x) in two independent experiments.

### Immunogold labeling

SKBR3 cells, cultured on 6-cm dishes, were washed in CO2-independent medium and incubated in CO2-independent medium containing 20μM GA, either alone or in the presence of proteasomal inhibitors 10μM MG132 and 10μM lactacystin for 2 hours at 37C. Cells were then prepared for cryo-immunoEM according to the Tokuyasu method. Briefly, cells were fixed in 2% paraformaldehyde - 0.2% glutaraldehyde in PBS for 2 hours at room temperature. Next, cells were gently scraped and embedded in 12% gelatin. After overnight Sucrose 2.3M infusion, small squared blocks were mounted on aluminium pins and frozen in liquid nitrogen. Ultrathin cryosections of 60nm were cut with Leica UltraCut UCT microtome (Leica Microsystems, Wetzlar, Germany) and immunolabeled with anti-ERBB2 antibody (9G6, Santa Cruz, St Louis, USA). Protein A-gold 15nm was used to reveal labeling of ERBB2.

### Immunofluorescence

For endocytic assays, TZ-Alexa488 was bound to SKBR3 cells at 4°C for 20 minutes before starting internalization for 2 hours at 37°C. Cells were than processed for immunofluorescence and labeled as described. Briefly, cells were fixed in 3% paraformaldheyde (PFA) in phosphate-buffered saline (PBS) pH 7.4 and then quenced with 30mM NH_4_Cl. After permeabilization with 0.2% saponin, cells were incubated with following antibodies. For co-localization studies under lysosomal inhibition, endogenous ERBB2 was detected with anti-ERBB2 9G6 (extracellular domain) antibody or internalized TZ (TZ-Alexa488), in combination with anti-EEA1 or LAMP-1 antibodies.

For co-localization studies under proteasomal inhibition, endogenous ERBB2 was detected with anti-ERBB2 9G6 (extracellular domain) antibody, used as primary antibody in combination with anti-ERBB2 Ab-1 (intracellular domain). The secondary antibodies were incubated for 20 min in 0.2% saponin/PBS: Alexa488 and Alexa546-conjugated donkey anti-mouse or donkey anti-rabbit IgG (Life Tecnhologies) and Alexa488-cojugated goat anti-human IgG (Molecular Probes). The coverslips were mounted using Prolong Gold with DAPI anti-fading reagent (Thermo Fisher Scientific). Image acquisition was perfomed with an Olympus IX70 epifluorescence microscope. Images were captured under oil with a 63x plan apochromat objective. When indicated, optical sections were acquired with TCS-SP2 AOBS confocal microscope station (Leica Microsystems, Wetzlar, Germany) or with an Axio Imager A2m equipped with an Apotome module for structured illumination epifluorescence (Carl Zeiss, Jena, Germany).

### Western blot and IP

SKBR3 whole cell lysates were prepared using EB Lysis Buffer (Hepes pH 7.4 20 mM, NaCl 150 mM, Glycerol 10%, Triton X-100 1%) with protease inhibitors cocktail (Roche, Basel, Switzerland) and sodium orthovanadate. Petri dishes were scraped to collect the whole lysates and incubated on ice for 20 minutes. Lysates were finally centrifuged for 2 minutes at 13200 rpm at 4°C to remove cellular debris. Protein cell extracts and SDS Polyacrylamide gel electrophoresis (NW04120box, BOLT Bis-Tris plus 4-12%, Invitrogen), were performed using standard protocols [61]. Proteins were detected with ECL Detection Reagent (Amersham, Little Chalfont Buckinghamshire, England). Protein quantification was performed using Bradford protein assay (BioRad, Hercules, CA, USA). ProteinA-Sepharose CL-4B (GE Healthcare, Piscataway, NJ, USA) was used for IP analysis. Rabbit anti-calnexin (sc-11397, Santa Cruz), rabbit anti-GAPDH (#2118, Cell Signaling) were used as loading control. Secondary antibodies were horseradish peroxidase-conjugated: anti-mouse (Molecular Probes, Thermo Fisher Scientific, Waltham, MA, USA), anti-rabbit (Molecular Probes) and anti-goat (Santa Cruz) and the detection of proteins were performed with ECL Detection Reagent (GE Healthcare).

### Statistical analysis

All parameters measured are presented as mean ± SEM and were analyzed with two-tailed Student's t-test or the nonparametric Mann–Whitney U test. P values <0.05 were considered statistically significant.

## SUPPLEMENTARY MATERIALS FIGURES AND TABLES


