# Supplementary material for: Identification of an HSP90 modulated multi-step process for ERBB2 degradation in breast cancer cells
Source: Oncotarget. 2016 Nov 16;7(51):85411–29. doi: 10.18632/oncotarget.13392 (PMC5356745; doi:10.18632/oncotarget.13392)
Supplement: Supplementary file 1 [file oncotarget-07-85411-s001.pdf]

Identification of an HSP90 modulated multi-step process for ERBB2 degradation in breast cancer cells

SUPPLEMENTARY FIGURES

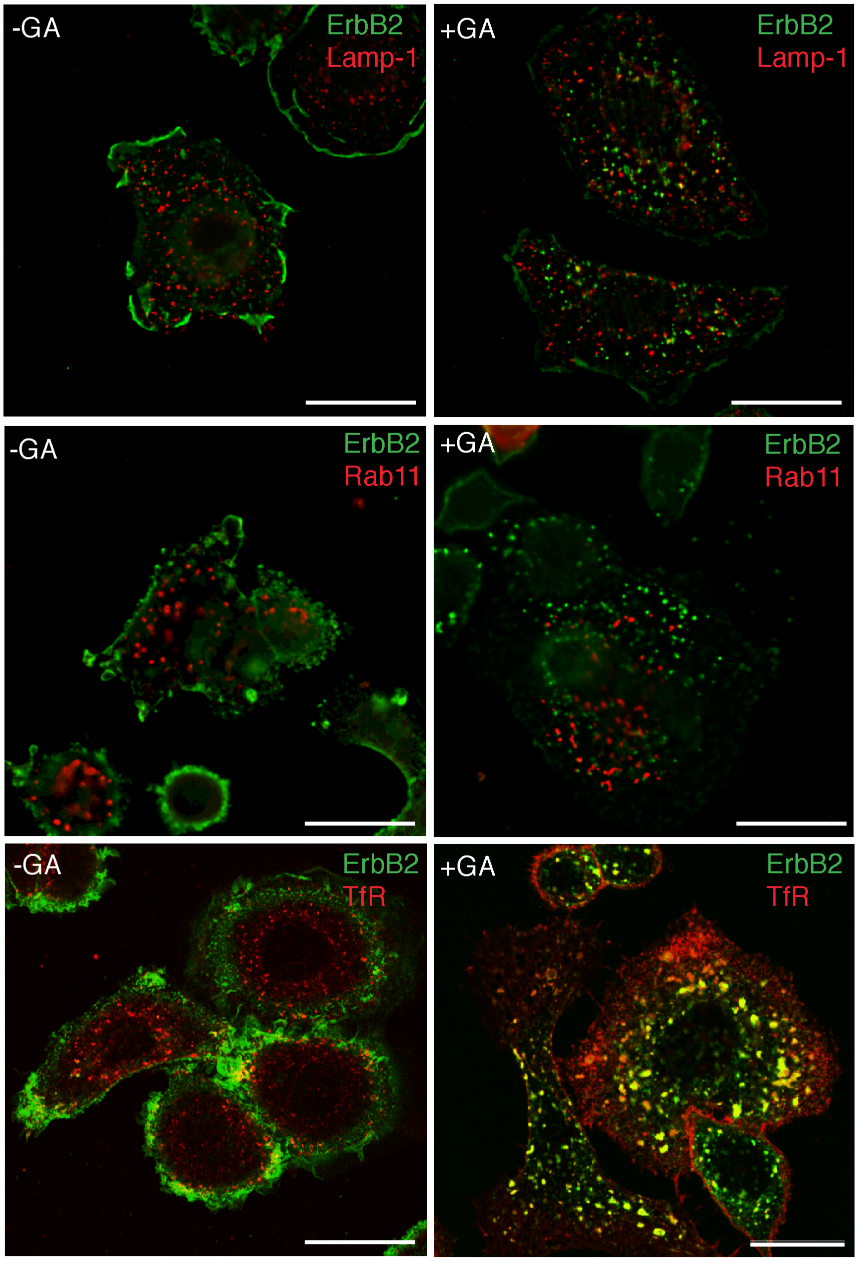

Supplementary Figure S1: Representative confocal images of SKBR3 cells in which ERBB2 (green signal, 9G6 antibody) was co-labeled with the lysosomal marker LAMP1 (red signal) in untreated and GA-treated cells (upper panel). Note that very few internalized ERBB2 is colocalizing in lysosomes when GA is added. In the lower panel, it is shown localization of ERBB2 (green signal, 9G6 antibody) in GA-treated cells for 2 hours at 37°C, transiently transfected wild-type DsRed-rab11 (left image, red signal) and endogenous transferrin receptor (right image, red signal). While colocalization (yellow signals) of ERBB2 with TfR can be clearly detected, no colocalization is seen with rab11-positive recycling endosomes and is negligible with LAMP1. Scale bar: 10 μM.

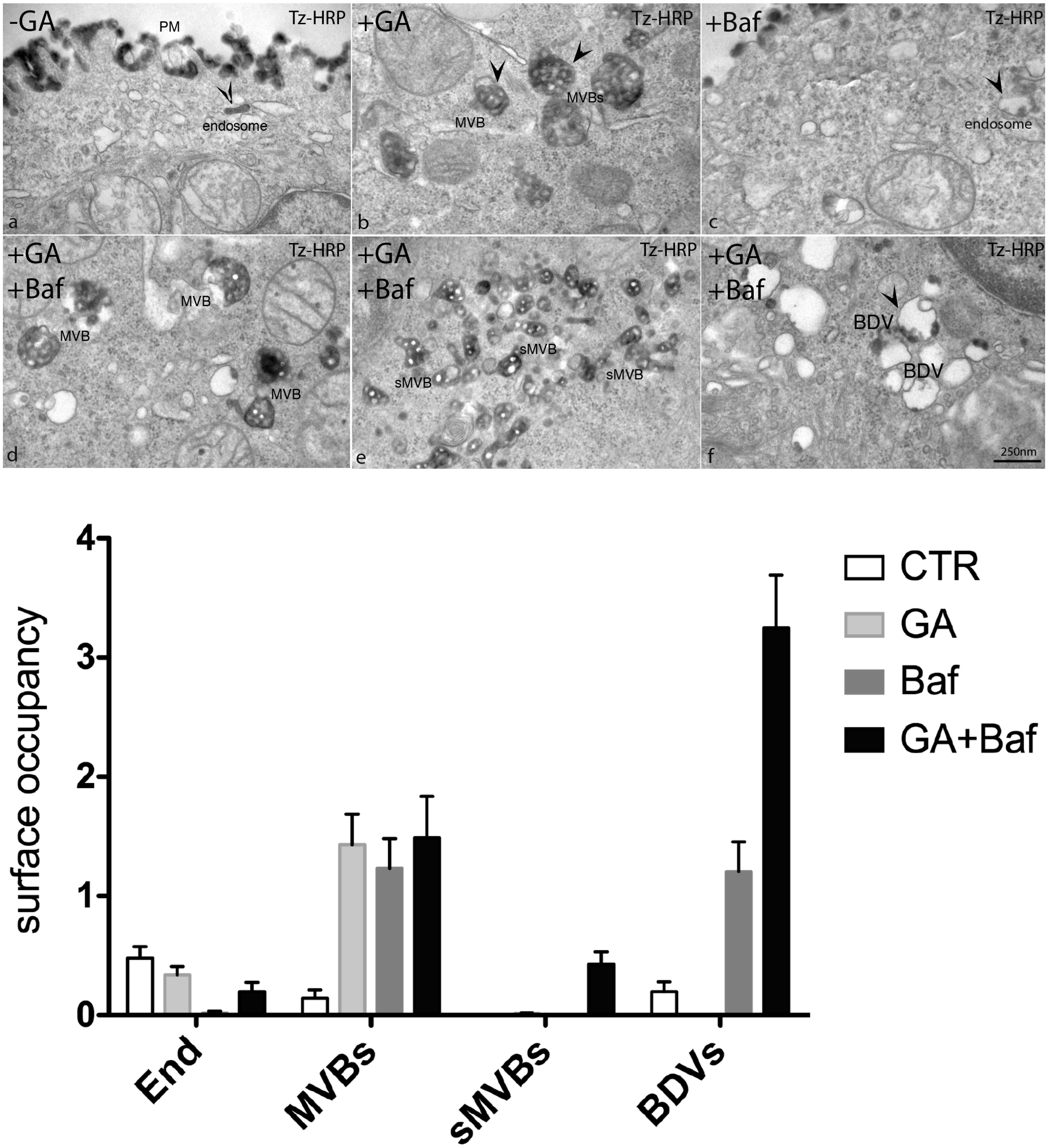

Supplementary Figure S2: Internalized ERBB2 accumulates within distinct MVBs populations and bafilomycin-derived vesicles (BDVs) upon lysosomal inhibition. A. Representative images of TZ-HRP positive endocytic structures in untreated SKBR3 cells (a), GA-treated (b) or BafilomycinA1 (Baf) treated cells (c) and co-treated with GA+Baf (d, e, f). HRP reaction product appears as a dark precipitate. (a) In control cells ERBB2 (TZ-HRP) is mainly localized at the PM. (b) After 2 hours of treatment with GA, internalized TZ-HRP is visible in MVBs. (c) Upon Baf treatment, TZ-HRP was found mainly at the plasma membrane. Three major categories of ERBB2-positive endocytic structures could be distinguished in GA+Baf co-treated cells: MVBs (d), small MVBs (e) and bafilomycin-derived vesicles (BDVs) (f). **B.** Histogram showing surface occupancy (mean±SEM) of each of the ERBB2-positive endocytic structures upon different treatments. Note that in co-treated (GA+Baf) cells the most represented category in which ERBB2 accumulates is BDVs, followed by small MVBs and MVBs. Scale bar: 250 nm.

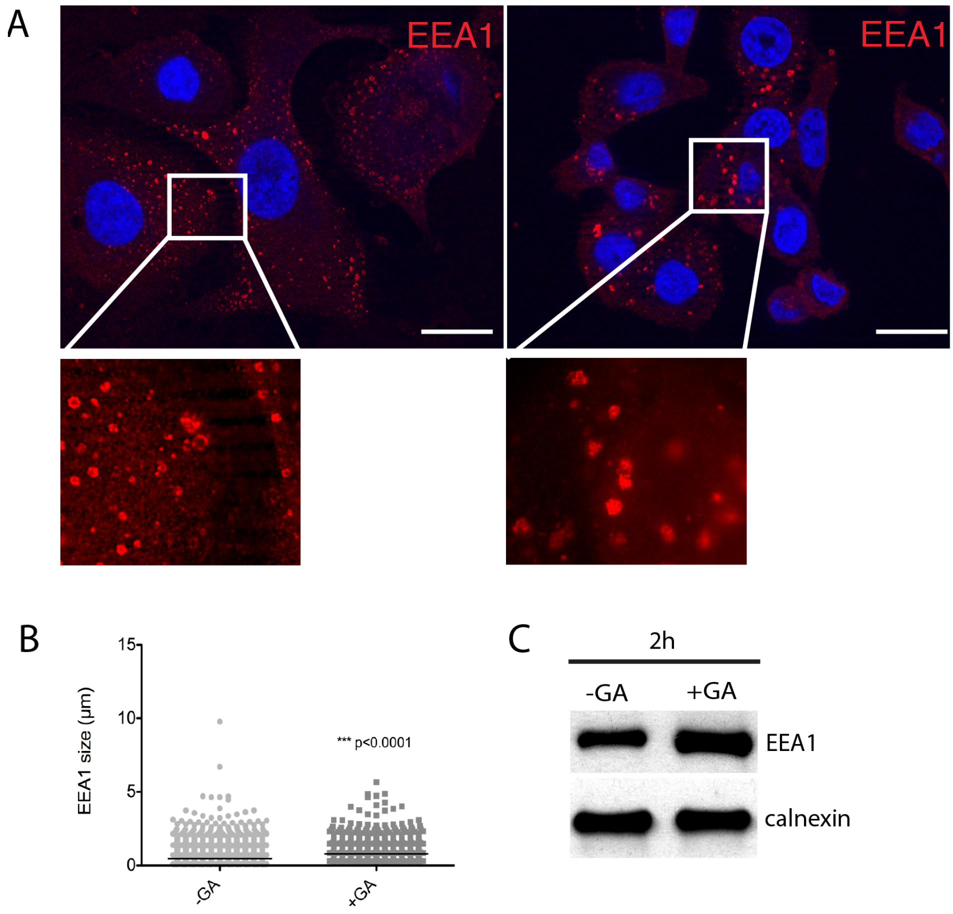

	-GA	+GA
Median	0.47	0.78

Supplementary Figure S3: GA alters early endosomes. A. Immunofluorescence images showing endogenous EEA1 labeling (red signal) in untreated and GA-treated cells for 2 hours at 37°C. Nuclei were stained with DAPI (blue signal). Note that in untreated cells EEA1-endosomes appear round, while in GA-treated cells they appear more irregular and with increased size (insets). **B.** The average size of EEA1-positive dots were calculated on 20 cells for each condition and plotted as scatter plot, (n=3). The line inside the scatter plot represents the median. In GA-treated cells, EEA1 endosomes were significantly larger than in untreated cells (0.47 vs 0.78 µm), ($p<0.001$). **C.** Western blot analysis of EEA1 total protein levels shows that GA increases the amount of EEA1 protein. Calnexin was used as loading control.

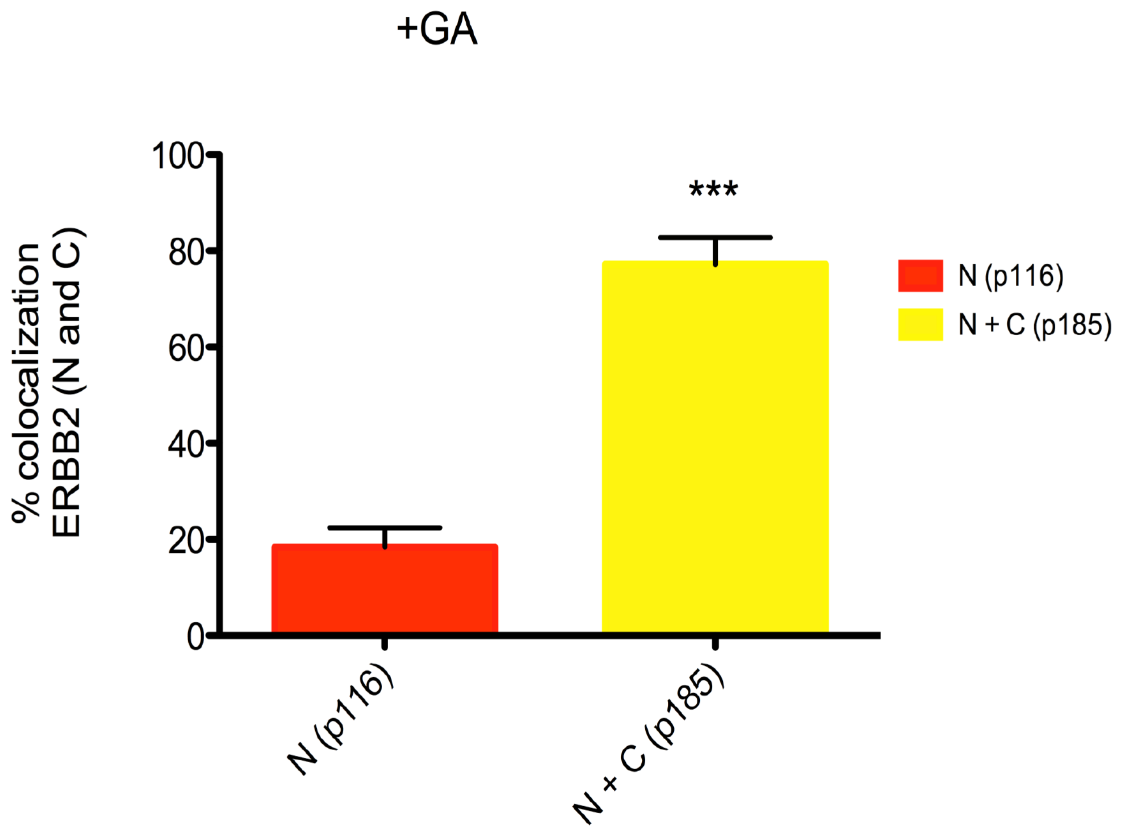

Supplementary Figure S4: Quantification of ERBB2 N-terminal and C-terminal signals colocalization of internalized ERBB2 upon GA treatment in SKBR-3 cells. Bar graph shows the quantification of internalized ERBB2 positive pixels calculated in SKBR3 cells treated with GA for 2h using the two antibodies directed against the N-terminal domain (N) (9G6, red channel) and the C-terminal domain (C) (Ab-1, green channel) of the receptor. The yellow bar shows the % of colocalization of the two antibodies (N+C, p185), the red bar represents the % of red pixels (N, p116) that did not colocalize with the green pixels. Bars show the mean, and error bars indicate the standard errors of the mean (SEMs) of pixels calculated on 15 images. *** $p<0.001$, Mann-Whitney test.

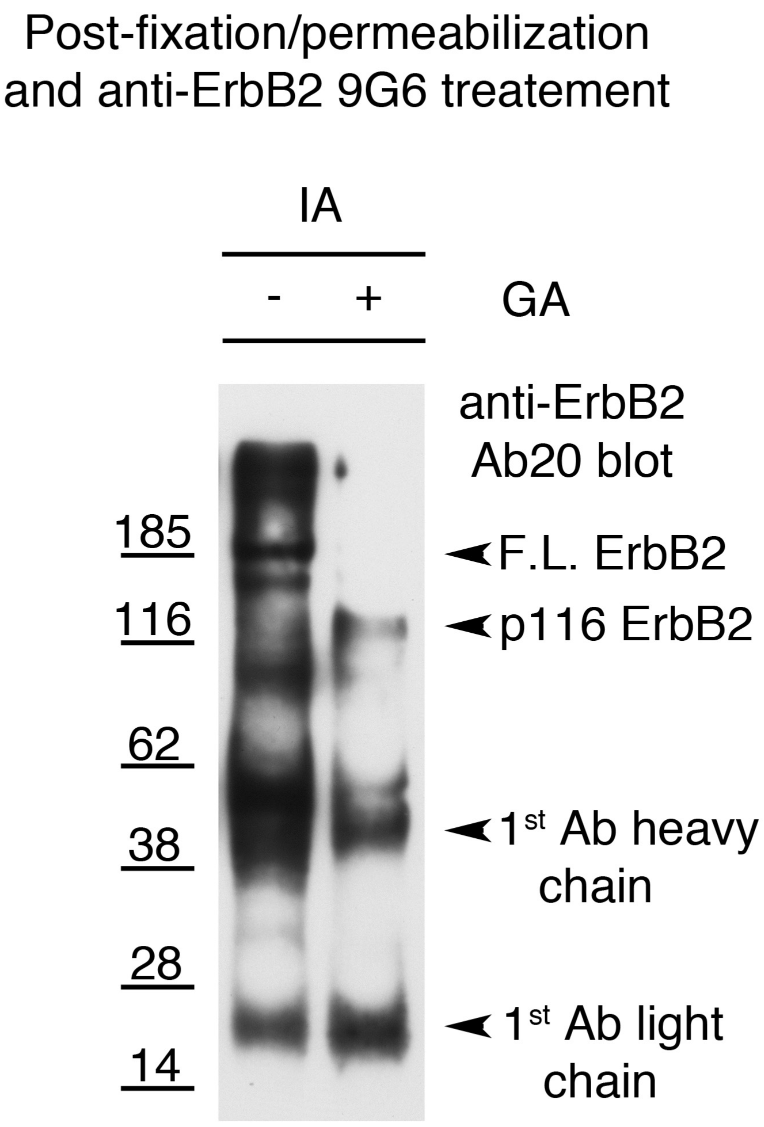

Supplementary Figure S5: The anti ERBB2 N-term specific antibody 9G6 recognizes the p116 ERBB2 isoform in experimental conditions used to perform immunofluorescence analysis. To verify that the anti ERBB2 N-term specific antibody 9G6 recognizes the p116 ERBB2 isoform in experimental condition used for immunofluorescence analysis, we cultured SKBR-3 in the presence or absence of GA for 2h and performed cell fixation, fluorescence quenching, permeabilization, 9G6 antibody binding and washes as described in the Materials and Methods section. Subsequentely, we performed cell lysis, as described in the Materials and Method section, and immunoaffinity purification (IA) on magnetic beads coated with Protein G (Bio Rad) following manufacturer's instructions. 9G6 immunocomplexes were eluted from protein G in SDS PAGE sample buffer. Samples were run in reducing conditions on SDS PAGE and blotted as described in the Materials and Methods section. The blotted membrane was challenged with the ERBB2 N-term specific antibody Ab 20, which was revealed by ECL as described in the Materials and Methods section. Protein standard markers are indicated by a line on the left along with their mass values expressed in kDa. On the right arrowheads indicate the migration of the: full length ERBB2 isoform (F.L. ERBB2); p116 ERBB2 isoform; 9G6 IgG heavy and light chains.

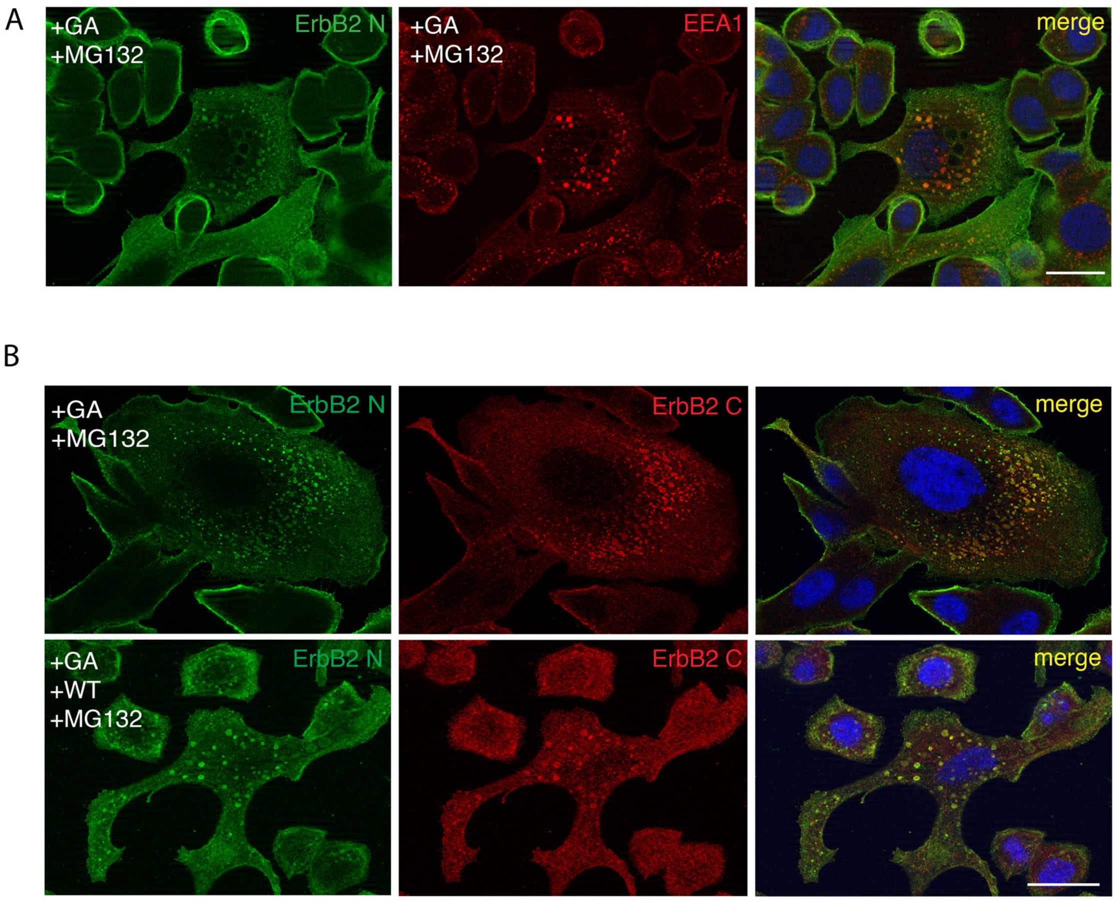

Supplementary Figure S6: Internalized ERBB2 is found in GA-altered EEA1-endosomes when cells are treated with MG132. A. Representative confocal microscopy images showing intracellular localization of ERBB2 N-terminal domain (green signal) and EE marker EEA1 (red signal) in GA+MG132 treated cells. **B.** Representative confocal microscopy images showing intracellular localization of ERBB2 N-terminal domain (green signal) and C-terminal domain (red signal) in GA+MG132 treated cells and GA+MG132+Wortmannin treated cells (lower panel). Note that both MG132 and wortmannin treatments greatly altered the size of EE. Size bar: 20µm.

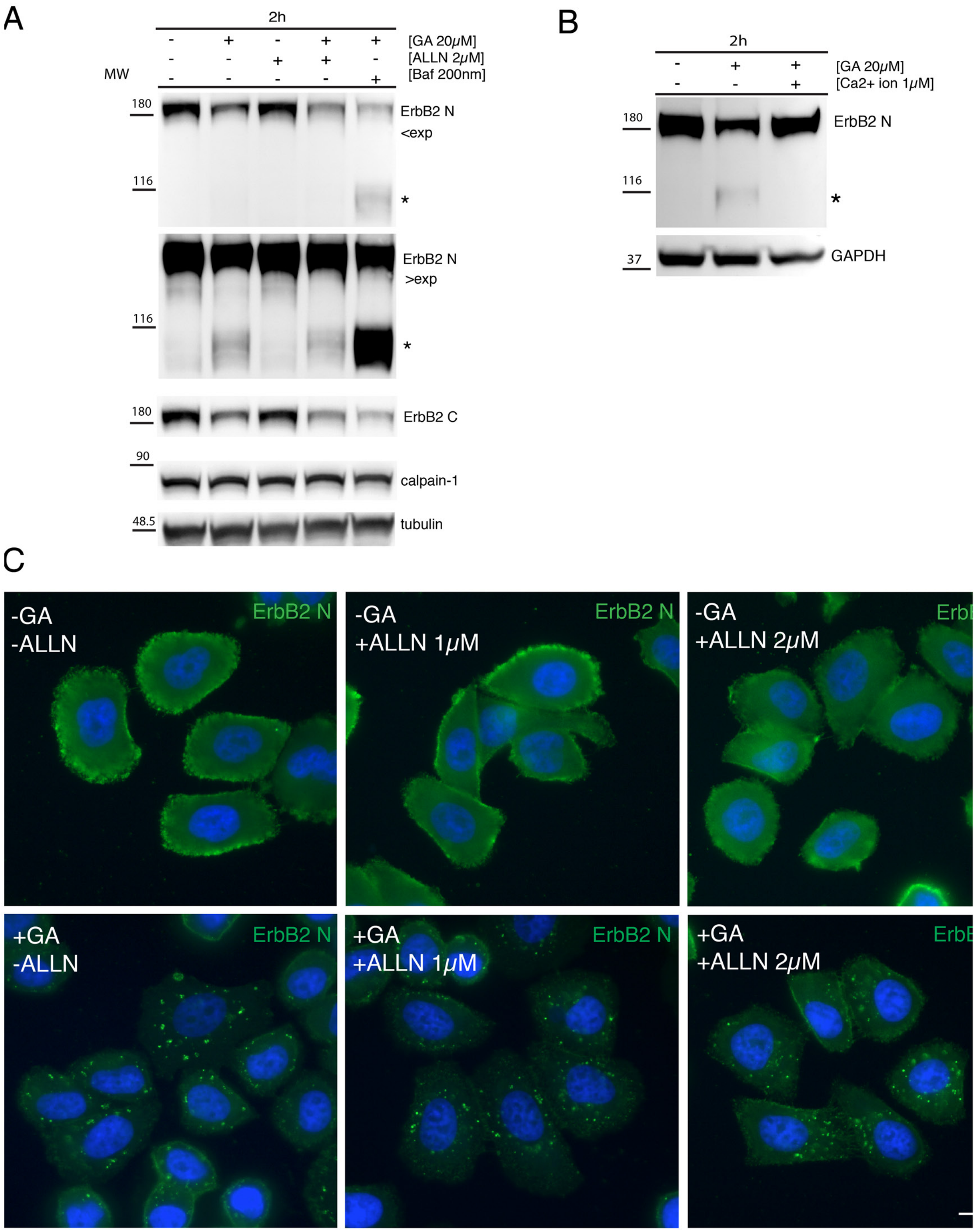

Supplementary Figure S7: Calpain-1 is not involved in GA-mediated cleavage of ERBB2. A. SKBR3 cells were untreated and treated with 20μM GA (lane 2), 2μM of the calpain inhibitor 1 (ALLN) (lane 3), and co-incubated with GA+ALLN (lane 4) or GA+Baf (lane 5) for 2 hours at 37°C. The total p185-ERBB2, the GA-induced p116-ERBB2, and calpain-1 protein levels were assessed by western blot using Abs recognizing the extracellular domain of ERBB2 (Ab-20), the intracellular domain of ERBB2 (C-18) and anti-calpain1 antibodies, respectively. Tubulin levels were used as loading control. Asterisks (*) indicate the p116 cleaved isoform of ERBB2. Two different exposures (exp) of the same blot are shown to better highlight the ERBB2 cleaved isoform, as indicated. **B.** SKBR3 cells were untreated and treated with 20μM GA (lane 2), 1μM Ca^{2+} ionophore (lane 3) to activate calpain-1 for 2 hours at 37°C. Note that the activation of calpain1 did not induce cleavage of ERBB2 (Ab-20). GAPDH was used as loading control. **C.** Representative immunofluorescence images of ERBB2 internalization (9G6, green signal) in the presence of 20μM GA and 1-2 μM ALLN for 2 hours 37°C, as indicated. DAPI (blue signal) was used to detect nuclei. On the left side of each panel the migration of protein molecular mass standards expressed in kDa is shown. Size bar: 10μM.

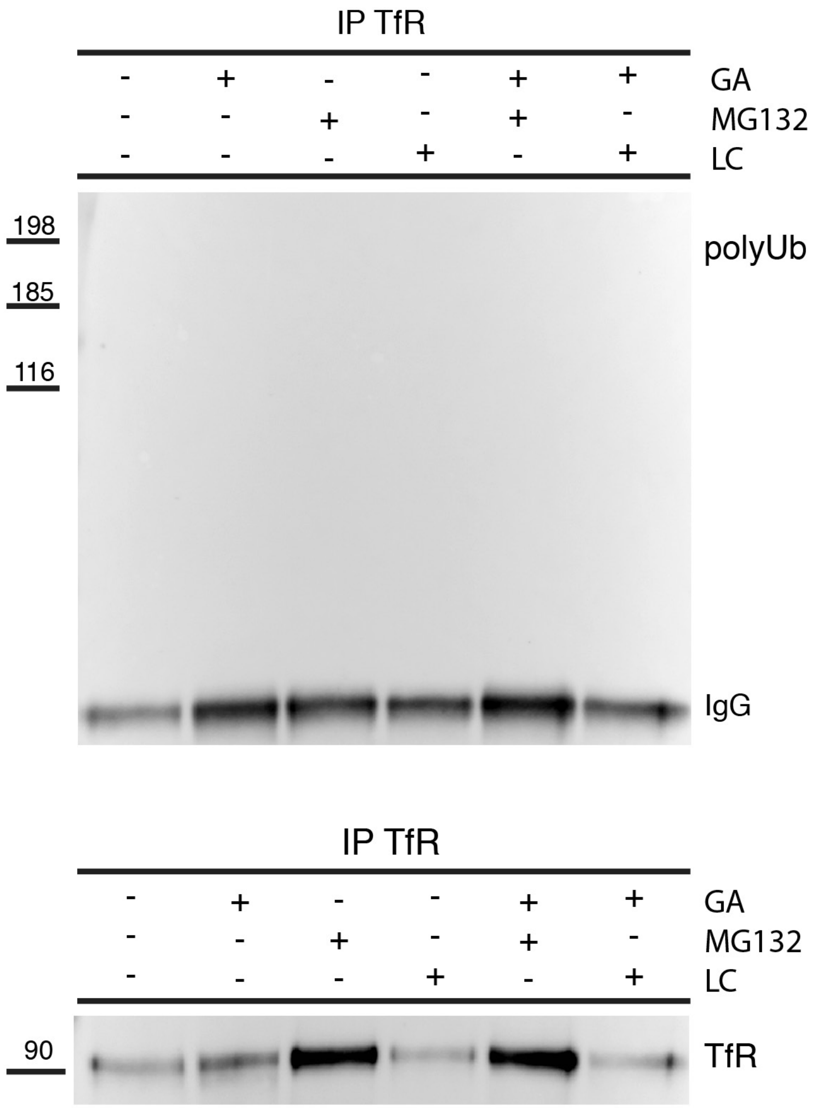

Supplementary Figure S8: Transferrin receptor is not polyubiquitinated under GA. SKBR3 cells were pre-treated with the proteasome inhibitors MG132 (10µM) and lactacystin (LC) (10µM) for 30 minutes at 37°C and GA was added for 1.5 hours at 37°C in the presence or absence of the inhibitors. Cells were then lysed as described under the material and method section. The cell lysates were immunoprecipitated with Transferrin receptor (TfR) as negative control for polyubiquitination. Immunoprecipitates (IP) were resolved by SDS/PAGE and immunoblotted with an anti-polyubiquitin (upper panel), and an anti-TfR antibody (lower panel) are shown. Note that TfR is not polyubiquitinated. On the left side of the panels the migration of protein molecular mass standards expressed in kDa is shown.

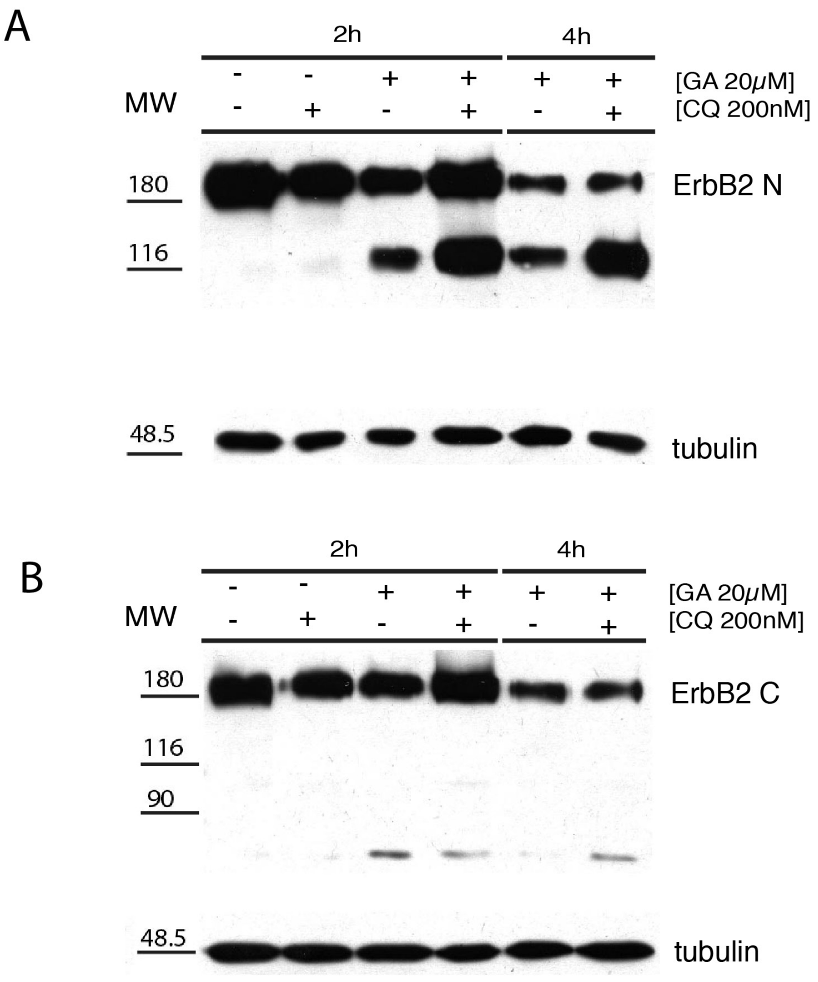

Supplementary Figure S9: ERBB2 cleavage and lysosomal degradation with cloroquine (CQ). SKBR3 cells were untreated or treated with 200nM CQ (lane 2), 20µM GA (lane 3-5), and co-incubated with GA+CQ (lane 4-6) for 2-4 hours at 37°C, as indicated. The total p185-ERBB2 and the GA-induced p116-ERBB2 protein levels were assessed by Western blot using Abs recognizing the extracellular domain of ERBB2 (Ab-20) (upper panel), and the intracellular domain (C-18), (lower panel). Levels of tubulin were used to confirm equal loading of proteins. On the left side of each panel the migration of protein molecular mass standards expressed in kDa is shown.

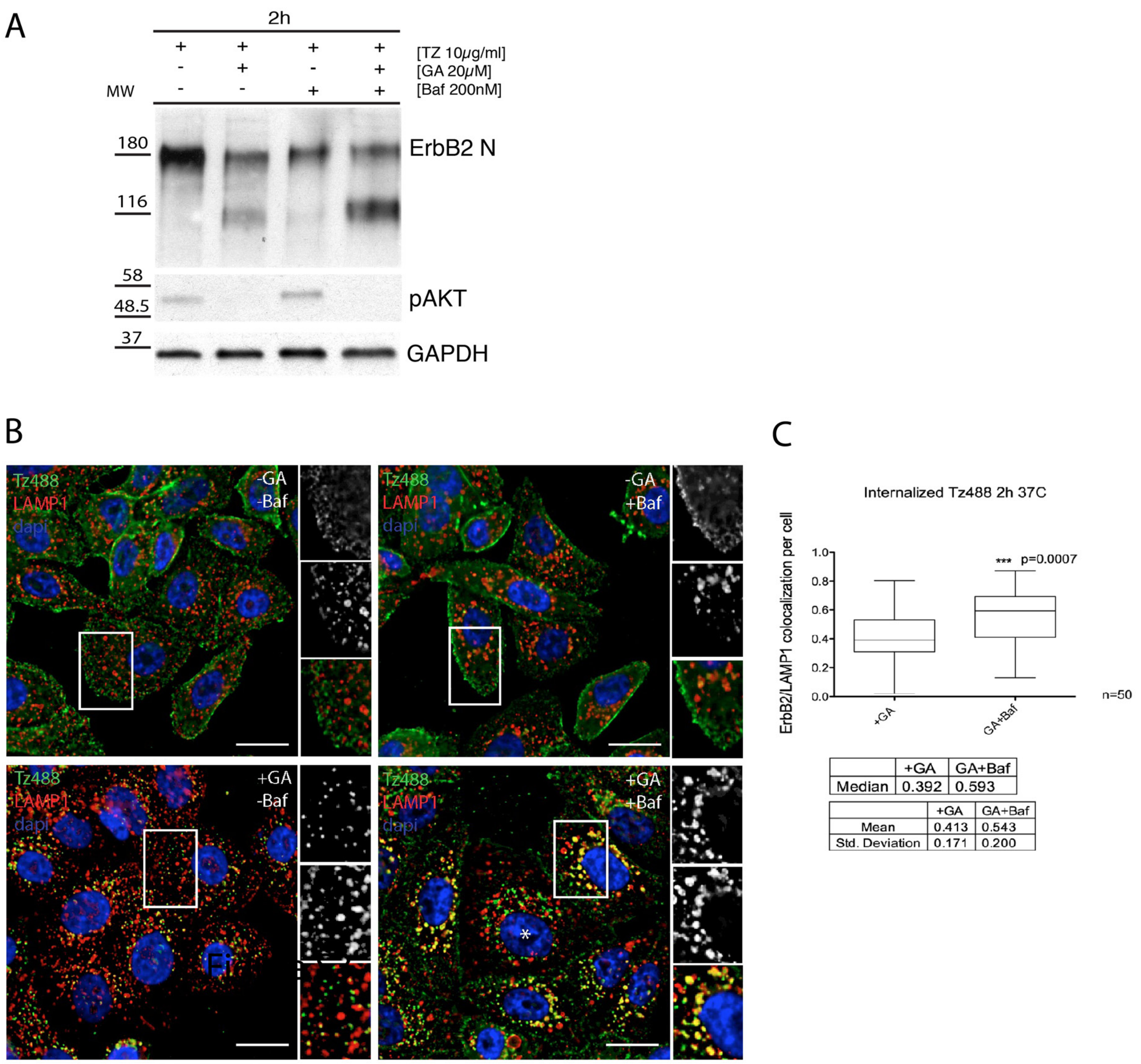

Supplementary Figure S10: TZ does not influence ERBB2 cleavage and trafficking to lysosomes. A. SKBR3 cells were untreated or treated with 20μM GA (lane 2), 200nM BafilomycinA1 (Baf) (lane 3), and co-incubated with GA+Baf (lane 4) for 2 hours at 37°C in the presence of 10μM TZ. The total p185-ERBB2, the GA-induced p116-ERBB2, and p-AKT protein levels were assessed by Western blot using an antibodies recognizing the extracellular domain of ERBB2 (Ab-20) and p-AKT. GAPDH was used as loading control. On the left side of each panel the migration of protein molecular mass standards expressed in kDa is shown. **B.** SKBR3 cells were pre-incubated with 200nM Baf for 30 minutes at 37°C and then pre-bounded with 10μg/ml of TZ-Alexa488 for 15 minutes at 4°C. After washing the excess of TZ-Alexa488 (green signal), cells were subsequently incubated with 20μM GA, Baf or GA+Baf for 1.5 hours at 37°C. Cells were fixed and labeled with anti-LAMP1 antibody (red signal). Representative confocal microscopy images are shown. Even in the presence of TZ and GA, only few ERBB2 is found inside lysosomes, whereas the colocalization is greatly increased when Baf was added in co-treatment with GA. Size bars: 10μM. **C.** Pixel quantification of ERBB2-LAMP1 colocalization per cell (n=3, 50 cells analyzed each experiment) in GA versus GA+Baf treated cells in the presence of TZ is depicted as box plot. The bottom and the top of each box are the first and third quartile, while the line inside the box represents the median (second quartile). The ends of the wiskers represent the minimum and the maximum data value. A significant increase (p=0.0007) of ERBB2 colocalization within LAMP1 compartments is shown when Baf inhibited lysosomal activity. Images and quantification are representative of three independent experiments.
